# Clinical study and some molecular features of Mexican patients with syndromic craniosynostosis

**DOI:** 10.1002/mgg3.1266

**Published:** 2020-06-08

**Authors:** Aurora Ibarra‐Arce, Manuel Almaraz‐Salinas, Víctor Martínez‐Rosas, Gabriela Ortiz de Zárate‐Alarcón, Laura Flores‐Peña, Mirza Romero‐Valdovinos, Angélica Olivo‐Díaz

**Affiliations:** ^1^ Departamento de Biología Molecular e Histocompatibilidad Hospital General “Dr. Manuel Gea González” Ciudad de México México; ^2^ División de Genética Hospital General “Dr. Manuel Gea González” Ciudad de México México

**Keywords:** craniosynostosis, *FGFR* genes, genetic variants, *TWIST1*

## Abstract

**Background:**

Craniosynostosis is one of the major genetic disorders affecting 1 in 2,100–2,500 live newborn children. Environmental and genetic factors are involved in the manifestation of this disease. The suggested genetic causes of craniosynostosis are pathogenic variants in *FGFR1*, *FGFR2*, *FGFR3*, and *TWIST1* genes.

**Methods:**

In order to describe their major clinical characteristics and the presence of pathogenic variants, a sample of 36 Mexican patients with craniosynostosis diagnosed as: Crouzon (OMIM 123,500), Pfeiffer (OMIM 101,600), Apert (OMIM 101,200), Saethre‐Chotzen (OMIM 101,400), and Muenke (OMIM 602,849) was analyzed.

**Results:**

In addition to craniosynostosis, most of the patients presented hypertelorism, midface hypoplasia, and abnormalities in hands and feet. To detect the pathogenic variants p.Pro252Arg *FGFR1* (OMIM 136,350), p.Ser252Trp, p.Pro253Arg *FGFR2* (OMIM 176,943), p.Pro250Arg, *FGFR3* (OMIM 134,934), and p.Gln119Pro *TWIST1* (OMIM 601,622), PCR amplification and restriction enzyme digestion were performed. Four and two patients with Apert presented the pathogenic variants p.Ser252Trp and p.Pro253Arg in *FGFR2*, respectively (with a frequency of 11.1% and 5.5%). The p.Pro250Arg pathogenic variant of *FGFR3* was found in a patient with Muenke (with a frequency of 2.8%). The above percentages were calculated with the total number of patients.

**Conclusion:**

The contribution of this work is discreet, since only 4 genes were analyzed and sample size is small. However, this strategy could be improved by sequencing the *FGFR1*, *FGFR2*, *FGFR3*, and *TWIST1* genes, to determine different pathogenic variants. On the other hand, it would be important to include other genes, such as *TCF12* (OMIM 600,480), *MSX2* (OMIM 123,101), *RAB23* (OMIM 606,144), and *EFNB1* (OMIM 300,035), to determine their participation in craniosynostosis in the Mexican population.

## Graphical abstract

We describe the major clinical characteristics of Mexican patients with craniosynostosis. Analysis of *FGFR1*, *FGFR2*, *FGFR3*, and *TWIST1* genes revealed a higher frequency of variants in Apert.

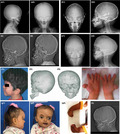

## 1 INTRODUCTION

Craniosynostosis describes partial or complete premature fusion of the cranial sutures, and occurs as part of a syndrome or as an isolated defect (nonsyndromic); the prevalence of craniosynostosis is 1 in 2,100–2,500 live births and is a significant cause of morbidity (Boulet, Rasmussen, & Honein, 2008). The cause, presentation, and management of craniosynostosis are heterogeneous. It may occur due to genetic variants or be result of mechanical, environmental, and hormonal factors during pregnancy.

Syndromic cases comprise 15%–30% and the rest are nonsyndromic. Specific single‐gene variants or chromosome abnormalities can be identified in at least 20% of all cases (Johnson & Wilkie, 2011; Kimonis, Gold, Hoffman, Panchal, & Boyadjiev, 2007).

It is known that *FGFR2* (176,943), *FGFR3* (134,934), *FGFR1* (136,350), *TWIST1* (601,622), and *EFNB1* (300,035) contribute significantly to the craniosynostosis‐associated syndromes. Pathogenic variants in *FGFR2* have been related to the Apert (AS) (OMIM 101,200), Pfeiffer (PS) (OMIM 101,600), Crouzon (OMIM 123,500), and Antley‐Bixler (OMIM 201,750) syndromes, and *FGFR3* have been related to the Muenke syndrome (MS) (OMIM 602,849), however, pathogenic variants in other *FGFR* genes may overlap between different syndromes.

*FGFR1*, *FGFR2,* and *FGFR3* belong to the family of fibroblast growth factor receptors (FGFR) (Itoh & Ornitz, 2004). Signaling in these genes plays a vital role in early embryonic development because they regulate the balance of cell proliferation. Differentiation and apoptosis are essential for the normal formation of cranial bones (Liu, Cui, Luan, Zhou, & Han, 2013). There are four types of FGFR, called tyrosine kinase receptors, and the pathogenic variants that affect these receptors allow ligand‐independent constitutive activation (functional gain), which leads to a premature ossification of the cranial sutures (Bonaventure & El Ghouzzi, 2003).

In turn, *TWIST* contains a basic helical loop helix (bHLH) motif, whose gene product acts as a transcription factor. The helix‐loop‐helix (HLH) region of this motif is important for homo‐ or heterodimerization, whereas the basic domain is essential for binding of the dimer complex to a target DNA‐binding sequence(s) (Paznekas et al., 1998). Several *TWIST* (OMIM 601,622) pathogenic variants related to Saethre‐Chotzen syndrome (OMIM 101,400) (SCS) have been described (El Ghouzzi et al., 1997; Howard et al., 1997).

Although craniosynostosis has been extensively studied worldwide, in Mexico it has only been addressed from the clinical diagnosis perspective, by identifying the mayor clinical syndromic features, and from the craniofacial surgery approach.

However, from the genetic perspective, few reports have been published of Mexican patients. For instance, there is one report of Jacobsen syndrome (JBS, OMIM 147,791), in which the author performs the karyotype and SNP array analysis in one JBS case. The patient presented developmental delay, craniosynostosis (dolichocephaly detected at 2 months of age), craniofacial dysmorphism, and a normal count and size of thrombocytes (Linares et al., 2015). Another report refers two unrelated patients of Crouzon with acanthosis nigricans (CAN OMIM 100,600) which presented an Ala391Glu pathogenic variant, specific of this form of the disease (Arnaud‐López, Fragoso, Mantilla‐Capacho, & Barros‐Nuñez, 2007). The third Mexican study was conducted by our group, focusing only on five patients with AS who underwent the molecular study of the *FGFR2* gene (Ibarra‐Arce et al., 2015).

Thus, the goal of this study was to describe the major clinical features associated with craniosynostosis, as well as to identify the frequency of pathogenic variants in *FGFR1*, *FGFR2*, *FGFR3,* and *TWIST1* in a sample of Mexican patients.

## 2 MATERIALS AND METHODS

### 2.1 Ethical compliance

This study was approved by the Ethics in Research and Research Committees of the Hospital General “Dr. Manuel Gea González”, with register number 10‐02‐2013. Written informed consent was obtained from each person or legal representative of the patients.

### 2.2 Clinical evaluation

In the period of 2008 to 2011, 36 patients with syndromic craniosynostosis were included, regardless of age and gender Medical Geneticists performed a complete clinical examination to give an accurate diagnosis of the patients, based on the clinical guidelines recommendations (McCarthy et al., 2012). Only patients with complete clinical features were considered. The cases were classified as Crouzon, AS, MS, PS, or SCS. Molecular results of five AS patients were previously described elsewhere (Ibarra‐Arce et al., 2015), but clinical characteristics are defined here. Since the clinical diagnosis is not really clear in some patients, we decided to include Crouzon patients in the molecular analysis, as a strategy to define if they were misclassified.

### 2.3 Pathogenic variant detection

DNA was extracted from EDTA‐peripheral blood using the proteinase K and phenol/chloroform extraction methods (Green & Sambrook, 2017). The following pathogenic variants in *FGFR1* (136,350) exon IIIa (NM_015850.3), *FGFR2* (176,943) exon IIIa (NM_000141.4), *FGFR3* (134,934) exon 7 (NM_000142.4), and *TWIST1* (601,622) exon 1 (NM_001002926.1) genes were selected, since they are important pathogenic variants in syndromic craniosynostosis: P252R, NM_023110.2(FGFR1):c.755C>G (p.Pro252Arg), rs121909627; S252W, NM_022970.3(FGFR2):c.755C>G (p.Ser252Trp), rs79184941; P253R, NM_000141.4(FGFR2):c.758C>G (p.Pro253Arg), rs77543610; P250R, NM_000142.4(FGFR3):c.749C>G (p.Pro250Arg), rs4647924; and Q119P, NM_000474.3(TWIST1):c.356A>C (p.Gln119Pro), rs104894057. The latter pathogenic variant was chosen because it alters the DNA‐binding domain modifying the affinity of *TWIST* to bind DNA (Howard et al., 1997).

To identify the specific pathogenic variant present in the patients, PCR amplification was performed in all patients of craniosynostosis for every pathogenic variant, independently of the syndrome. Primers and restriction enzymes used are listed in Table 1 (Bellus et al., 1996; Graham et al., 1998; Howard et al., 1997; Lajeunie et al., 2006; Meyers et al., 1996; Pandey, Bajpai, Ali, Gayan, & Singh, 2013; Park et al., 1995; Paznekas et al., 1998; Zeiger et al., 2002).

**TABLE 1:** Primers and conditions for genomic amplification of *FGFR1, 2, 3, and TWIST1*

Gene	Primers 5′‐3′	Amplicon size (bp)	Restriction enzyme	Digested fragments (bp)
*FGFR1* exon IIIa	Fw: AAGTGCCTCCTCTCCCATCTTC Rev: TGAACTCCACGTTGCTACCCAG	216	*MnlI*	136, 109 (Lajeunie et al., 2006; Pandey et al., 2013; Zeiger et al., 2002)
*FGFR2* exon IIIa	Fw: TGACAGCCTCTGACAACACAAC Rev: GGAAATCAAAGAACCTGTGGC	350	*BstXI*	212 and 141 (Meyers et al., 1996; Pandey et al., 2013; Park et al., 1995)
*FGFR3* exon 7	Fw: CGGCAGTGACGGTGGTGGTGAG Rev: CCAAATCCTCACGCAACCC	341	*NciI*	151, 123, 67 (Bellus et al., 1996; Graham et al., 1998; Paznekas et al., 1998)
*TWIST 1* exon 1	Fw: GAGGCGCCCCGCTCTTCTCC Rev: AGCTCCTCGTAAGACTGCGGAC	378	*BstUl*	22, 31, 35, 53, 63, 98 and 210 (Howard et al., 1997; Paznekas et al., 1998)

Note

*FGFR1* ex IIIa (136,350) Pfeiffer (P252R;136,350.0001), rs121909627; *FGFR2* exon IIIa (176,943) Apert (101,200)(S252W;176,943.0010), rs79184941; *FGFR2* exon IIIa (176,943) Apert (101,200) (P253R;176,943.0011), rs77543610; *FGFR2* exon IIIa (176,943) Crouzon (123,500)(T268TG9); *FGFR3* exon 7 (134,934) Muenke (602,849) (P250R;134,934.0014), rs4647924; *TWIST1* ex 1 (601,622) Saethre‐Chotzen (101,400)( GLN119PRO;601,622.0002), rs104894057.

The optimized PCR conditions consisted of 200 ng of DNA, 0.5 mmol/L of each primer, 0.2 mmol/L dNTPs, PCR buffer (10 mmol/L Tris‐HCl, pH 8.3; 50 mmol/L KCl), 1.5 mmol/L MgCl_2_, and 2 U Taq polimerase (Promega, Madison, WI, USA). The PCR carried out under the following conditions: 35 cycles of denaturation at 95°C for 1 minute, annealing at 60–62°C for 1 minute, and extension at 72°C for 1 minute. Amplicons were verified by 2% agarose gel electrophoresis.

Pathogenic variants were detected by specific restriction enzyme digestion of each amplicon, performing the reaction according to the manufacturer's instructions (New England Biolabs, Ipswich, MA, USA), and subsequently analyzed on a 6.0% polyacrylamide gel.

We used the HGVS (Human Genome Variation Society) recommendations for the description of the pathogenic variants (den Dunnen et al., 2016) and the access number for the OMIM genes was included.

## 3 RESULTS

### 3.1 Baseline characteristics

Age range of the 36 patients was from 6 to 44 years, and the average maternal and paternal age was 31.4 years (from 20 to 43 years) and 35.1 years (from 25 to 55 years) at the time of the patient's birth, respectively. Seven Crouzon had a family history of craniosynostosis: one with father and brother with Crouzon; one with mother and cousin with Crouzon; one with mother and sister with Crouzon; one with grandmother and aunt with Crouzon; one with grandfather with ocular proptosis; one with a brother and cousin referred only as craniosynostosis; and one with a daughter with Crouzon.

The main clinical features that the patients presented, in addition to craniosynostosis, were the following: hypertelorism, midface hypoplasia, and abnormalities in hands and feet; different skull forms were observed.

Table 2 presents 41 clinical and molecular characteristics of the 5 craniosynostosis syndromes studied making a comparison with the literature.

**TABLE 2:** Main characteristics of the patients with craniosynostosis

Characteristics	Ciurea and Toader (2009)	Wilkie et al. (2010)	Johnson and Wilkie (2011)	Agochukwu et al. (2012)	Palafox et al. (2012)	Twigg and Wilkie (2015)	Ko (2016)	Kutkowska‐Kaźmierczak et al. (2018)	This study (%)					
									AS (*n* = 6)	MS (*n* = 1)	PS (*n* = 4)	SCS (*n* = 4)	Crouzon (*n* = 18)	MD (*n* = 3)
Acrocephalus	AS	NA	NA	NA	AS	NA	NA	AS	100	0	0	0	5.6	0
Brachycephalus	Crouzon	NA	NA	NA	NA	MS	PS	Crouzon, PS, SCS	0	100	25	25	66.7	66.7
Dolico/scaphocephalus	NA	NA	NA	NA	NA	NA	NA	NA	0	0	25	0	5.6	0
Turricephalus	NA	NA	NA	NA	NA	NA	NA	NA	0	0	25	0	11.1	33.3
Plagiocephalus	NA	NA	NA	NA	NA	NA	NA	NA	0	0	25	75	0	0
Acrobrachyturricephalus	NA	NA	NA	NA	NA	NA	NA	NA	0	0	0	0	11.1	0
Hypertelorism	Crouzon	Crouzon	NA	NA	NA	NA	AS	Crouzon, AS, PS, SCS	100	0	50	0	27.8	66.7
Ocular proptosis	Crouzon	Crouzon	NA	Crouzon	Crouzon, AS	Crouzon, SCS	Crouzon, AS, PS, MS, SCS	Crouzon, AS, PS	100	100	50	50	100	66.7
Palpebral ptosis	SCS	Crouzon	NA	SCS	SCS	SCS	SCS	Crouzon, SCS	17	0	0	50	16.7	33.3
Strabismus	Crouzon	NA	NA	NA	Crouzon	NA	NA	Crouzon, AS	33	0	0	25	50	33.3
Nistagmus	NA	NA	NA	NA	NA	NA	NA	NA	0	0	25	0	0	0
Choanal stenosis or choanal atresia	NA	NA	NA	NA	NA	NA	NA	NA	33	0	0	0	0	0
Midface hypoplasia	Crouzon, AS, PS, MS, SCS	Crouzon, AS	MS	Crouzon, AS, PS	Crouzon, AS, SCS	Crouzon, AS	AS, PS	Crouzon, PS, SCS	100	100	75	50	77.8	66.7
Beaked nose	Crouzon	Crouzon	NA	NA	NA	Crouzon	NA	Crouzon	0	0	0	0	27.8	33.3
Malocclusion	NA	NA	NA	NA	Crouzon	NA	AS	Crouzon	17	0	25	0	72.2	33.3
Prognathism	Crouzon	NA	NA	NA	NA	NA	NA	Crouzon	0	0	25	0	44.4	66.7
Retrognathia	NA	NA	NA	NA	NA	NA	NA	NA	0	0	0	25	5.6	0
High ogival/palate	NA	NA	NA	NA	NA	NA	NA	Crouzon	17	0	25	0	33.3	0
Cleft lip palate	SCS	NA	AS	AS	NA	NA	AS	AS, SCS	17	0	25	25	5.6	0
Ears with low implantation	NA	NA	NA	NA	AS	NA	NA	NA	0	0	0	0	38.9	33.3
Hearing loss	NA	NA	NA	NA	Crouzon	NA	AS	AS	17	0	0	0	5.6	33.3
Otitis media	NA	NA	NA	NA	NA	NA	NA	AS	100	0	0	25	0	0
Short neck	NA	NA	NA	NA	NA	NA	NA	NA	33	0	25	0	44.4	
Hands syndactyly	A, SCS	AS	AS, SCS	AS, SCS	AS, SCS	AS	AS	AS, SCS	100	0	75	50	0	66.7
Broad first toe in hands	NA	NA	NA	NA	NA	NA	NA	NA	0	100	0	0	5.6	33.3
Feet syndactyly	NA	NA	NA	NA	NA	NA	NA	NA	33	0	0	0	0	33.3
Broad first toe in feet	NA	NA	SCS	NA	PS	NA	NA	NA	33	0	50	25	11.1	33.3
Geno valgus	NA	NA	NA	NA	NA	NA	NA	NA	0	0	0	25	16.7	0
Rhizomelic shortening	NA	NA	NA	NA	NA	NA	NA	NA	83	0	0	0	0	0
Glenohumeral dysplasia	NA	NA	NA	NA	NA	NA	NA	NA	50	0	0	0	0	0
Elbow ankylosis	NA	NA	NA	NA	NA	NA	NA	NA	50	100	0	0	0	0
Hydrocephalus	NA	NA	NA	NA	NA	NA	NA	AS	33	0	0	0	0	0
Agenesis of the corpus callosum	NA	NA	NA	NA	AS	NA	NA	AS	50	0	0	0	0	0
Heart disease	NA	NA	NA	NA	NA	NA	NA	NA	50	0	0	0	0	0
Hyperhidrosis	NA	NA	NA	NA	NA	NA	NA	AS	83	0	0	0	0	0
Autism	NA	NA	NA	NA	NA	NA	NA	NA	0	0	0	0	5.6	0
Speech disorders	NA	NA	NA	NA	NA	NA	NA	NA	17	0	0	0	5.6	0
Developmental delay	NA	NA	NA	NA	NA	NA	NA	AS	33	100	0	0	11.1	0
Intellectual disability	AS, PS	NA	AS	NA	AS	NA	AS	NA	17	0	25	0	5.6	0
Family history	NA	NA	NA	NA	NA	NA	NA	NA	0	0	0	0	38.9	0
Pathogenic variant														
*FGFR2* (p.Ser252Trp)*	AS	NA	AS	NA	NA	NA	AS	AS	67	0	0	0	0	0
*FGFR2* (p.Pro253Arg)**	AS	NA	AS	NA	NA	NA	AS	AS	33	0	0	0	0	0
*FGFR3* (p.Pro250Arg)***	MS	NA	MS	MS	MS	MS	MS	NA	0	100	0	0	0	0

Abbreviations: ***NM_000142.4(FGFR3):c.749C>G (p.Pro250Arg); **NM_000141.4(FGFR2):c.758C>G (p.Pro253Arg); *NM_022970.3(FGFR2):c.755C>G (p.Ser252Trp); AS, Apert syndrome; Crouzon, Crouzon Syndrome; MD, Misdiagnosed; MS, Muenke syndrome; NA, Not applicable; PS, Pfeiffer syndrome; SCS, Saethre‐Chotzen 0syndrome.

**FIGURE 1:**
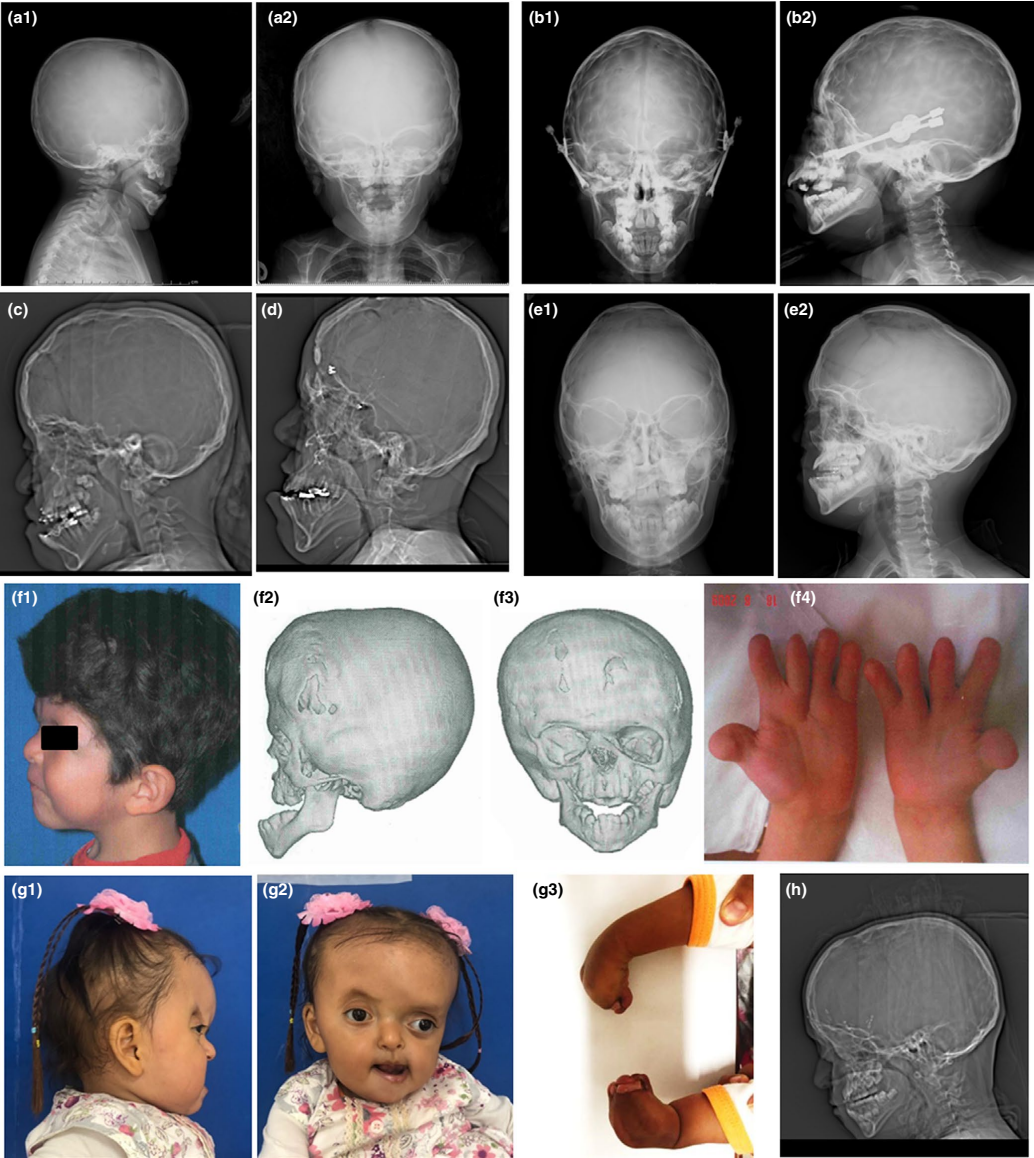
(a) Two‐year‐old male patient with Apert syndrome (AS‐5), skull radiography study in lateral (a.1) and anteroposterior (a.2) projection, showing: coronal synostosis, widening of the sagittal suture, retrusion of the midface hypoplasia, shallow orbits, digital printing at the temporoparietal level, and decrease in anteroposterior diameter. (b) Seven‐year‐old male patient with Crouzon syndrome, postoperative, with anteroposterior (b.1) and lateral (b.2) skull radiography. Findings: remodeling with widespread digital impressions suggestive of intracranial hypertension, distracting of the middle facial third. (c) Ten‐year‐old female patient with Crouzon syndrome, image of the lateral topography where it is observed: decrease in the anteroposterior diameter with a discreet increase in the craniocaudal diameter, digital impression, and retrusion of the midface hypoplasia. (d) Twenty‐six‐year‐old male patient with Apert syndrome (AS‐1), lateral topogram image showing: anteroposterior decline, postorbital frontal advance change, and severe retrusion of the midface hypoplasia. (e) Eight‐year‐old male patient with Crouzon syndrome, with anteroposterior (e.1) and lateral (e.2) cranial radiography, showing: postorbital frontal advance changes, retrusion of the midface hypoplasia, with irregularities in the cranial vault. (f) Eight‐year‐old male patient with Apert syndrome (AS‐2), flat facial profile, well‐shaped atrial canopy with low implantation (f.1), with 3D reconstruction images of CT scan of the lateral (f.2) and anterior (f.3) skull, where a decrease in the anterior posterior diameter of the skull is observed, wide forehead. In the frontal image, asymmetric orbits are observed, with smaller size of the left orbit and displaced below it, associated with midface hypoplasia, plagiocephaly, and prognathism. In the hand (f.4), short fingers (brachidactyly) with surgical scar are observed by correction of syndactyly. It presents cutaneous syndactyly and first toe with lateral displacement. (g) Three‐year‐old female patient with Apert syndrome; lateral (g.1) and frontal (g.2) photography, coronal closure of the left side is observed; type III syndactyly is present in the hands (g.3). (h) Eight‐year‐old male patient with Crouzon syndrome; image of lateral skull topogram where changes are observed postadvance fronto‐orbital with harmonic anteroposterior diameter and retrusion of the middle facial third

The Crouzon patients (*n* = 18), all had ocular proptosis; midface hypoplasia 14; malocclusion 13; strabismus 9, prognathism 8; short neck 8; ears low implantation 7; hypertelorism 5; beaked nose 5; palpebral ptosis 3; geno valgus 3; broad first toe in feet 2; hearing loss 1; and broad first finger in the hand 1 (Table 2 and Figure 1b,c,e and h). None of the cases with clinical diagnosis of Crouzon syndrome had acanthosis nigricans.

The distinctive clinical characteristics of AS (*n* = 6), in addition to craniosynostosis, are acrocephaly, hypertelorism, midface hypoplasia, ocular proptosis, hand syndactyly, and otitis media; 5 cases presented rhizomelic shortening and hyperhidrosis; 3 elbow ankylosis, heart disease, and glenohumeral dysplasia; 3 agenesis of the corpus callosum; 2 strabismus; 2 choanal stenosis or choanal atresia; 2 hydrocephaly and developmental delay; 2 short neck; and 1 palpebral ptosis, speech disorders, and cleft/lip palate; and 1 malocclusion, hearing loss, and intellectual disability. In *FGFR2*, four patients presented the pathogenic variant p.Ser252Trp and two the pathogenic variant p.Pro253Arg, with a frequency of 11.1% and 5.5%, respectively (Table 2).

In MS (*n* = 1), the case had brachycephaly, ocular proptosis, midface hypoplasia, broad first toe in hands, geno valgus, elbow alteration, intellectual disability, and the p.Pro250Arg pathogenic variant in *FGFR3*. The frequency of the variant was 2.8%.

Four cases of type I PS (*n* = 4) were detected, each with a different type of skull: plagiocephalus, brachycephalus, dolico/scaphocephalus, and turricephalus. Three cases presented midface hypoplasia (patients 1, 2 and 4); 2 cases hypertelorism (patients 3 and 4); 2 ocular proptosis (patients 1 and 2); 2 broad first toe in feet (patients 1 and 4); 1 nistagmus, hearing loss and developmental delay (patient 2); 1 prognathism and cleft lip palate (patient 3); 1 high ogival palate (patient 1); and 1 mal occlusion (patient 4) (Table 2).

In SCS (*n* = 4), 3 cases showed plagiocephaly (patients 1, 3, and 4); 2 cases midface hypoplasia (patients 2 and 4); 2 ocular proptosis (patients 1 and 4); 2 palpebral ptosis and short neck (patients 1 and 3); 1 strabismus, syndactyly in feet, and broad first toe in feet (patient 3); 1 retrognathia (patient 4); 1 cleft/lip palate (patient 2); and 1 ears with low implantation (patient 1) (Table 2).

### 3.2 Clinical cases with pathogenic variants

AS‐1: Male patient of 26 years with AS, with no family history of craniosynostosis (with 1 normal brother), maternal age 40 years and paternal age 43 years, mother had a previous abortion (6 weeks), is a product of pregnancy 3, pregnancy with threatened abortion at 2 months, for which the mother was treated and the delivery was premature, at 7 months of gestation, and delivery with fetal distress. In addition to acrobrachycephaly, midface hypoplasia, hypertelorism, ocular proptosis, strabismus, high ogival/palate, otitis media, hearing loss, and syndactyly of the hands and feet of AS, it also presents vertebral fusion at cervical level and lordosis, heart problems with diastolic murmur, dilation of the right ventricle, tricuspid insufficiency, and aortic insufficiency; pulmonary stenosis, tetralogy of Fallot, hydrocephalus, symptomatic epilepsy, and seizures were present. He presented complete syndactyly type II in hands and type IV in the feet. The patient exhibited moderate intellectual disability, with developmental delay. This patient showed the p.Pro 253Arg pathogenic variant in *FGFR2* exon IIIa (Figure 1d).

AS‐2: Male patient of 8 years; maternal age 38 years, paternal age 37 years. Besides the craniofacial typical characteristics of AS, it presents heart disease, urogenital abnormality, type I syndactyly in hands, with language and movement problems. In this patient, the p.Ser252Trp pathogenic variant in *FGFR2* exon IIIa was present (Figure 1 from f.1 to f.4).

AS‐3: Female patient of 1 year, family history is unknown, maternal age 36 years, and paternal age 43 years. In hands presented type III syndactyly (fused fingers), grade I chronic malnutrition was also diagnosed. The patient had the p.Ser252Trp pathogenic variant in *FGFR2* exon IIIa.

AS‐4: Male patient of 1 year, family history is unknown; maternal age 20 years, without data of the father. With heart disease, type I syndactyly in hands, and the patient had the p.Ser252Trp pathogenic variant in *FGFR2* exon IIIa gene.

AS‐5: Male patient of 2 years, family history unknown, maternal age 25 years, paternal age 44 years. With type I syndactyly in hands and the p.Ser252Trp pathogenic variant in *FGFR2* exon IIIa (Figure 1, a.1 and a.2).

AS‐6: Male patient of 37 years, family history unknown, with type I syndactyly in hands, and the p.Pro253Arg pathogenic variant in *FGFR2* exon IIIa.

MS: Female patient of 2 years, maternal age 40 years, paternal age 42 years; with 4 healthy brothers; the mother had 2 previous abortions. The p.Pro250Arg pathogenic variant in *FGFR3* exon IIIa was present.

## 4 DISCUSSION

In this study, we describe the main clinical characteristics of 36 patients with craniosynostosis, as well as some of the pathogenic variants of *FGFR1, FGFR2, FGFR3,* and *TWIST1* genes.

As it is known, craniosynostosis has a wide clinical spectrum and the shape of the skull can help in the diagnosis, as well as in the surgical approach, together with other characteristics in the limbs.

When comparing the types of skulls that exist in the patients, different forms were observed, from a single skull type in AS (Acrocephalus), up to 5 different types in Crouzon: acrocephalus, brachycephalus, dolico/scaphocephalus, turricephalus, and acrobrachyturricephalus.

Multiple hypotheses have been proposed to explain the pathogenesis of abnormal fusion of sutures. Both environmental factors (especially the restriction of the intrauterine fetal head) and genetic factors (variants of a single nucleotide, chromosomal abnormalities, and polygenic background) predispose to craniosynostosis.

When the skull has an elongated and narrow shape, similar to a boat is called scaphocephaly (Massimi, Caldarelli, Tamburrini, Paternoster, & Di Rocco, 2012). Our patients with PS (1 of 4) and Crouzon (1 of 18) presented this characteristic. Unilateral coronal synostosis, resulting in a frontal plagiocephalic skull, is the second most common form of skull in patients with syndromic craniosynostosis, representing up to 25% of cases of craniosynostosis (Raposo‐Do‐Amaral et al., 2011; 2015), predominantly affects females (60%), with similar incidence on both sides of the skull. In our patients plagiocephaly was found in SCS (75%) and PS (25%). The premature fusion of the coronal suture also causes a deviation in the base of the skull, changing the position of the orbits, asymmetry of the eyebrows, asymmetry of the position of the ear, deviation of the jaw and change of occlusion, with an important effect aesthetic (Ghizoni, Raposo‐Amaral, Mathias, Denadai, & Raposo‐Amaral, 2013; Raposo‐Amaral, García, Denadai, Raposo‐Amaral, & Raposo‐Amaral, 2014; Raposo‐Amaral et al., 2013).

Strabismus is a general finding in patients with syndromic craniosynostosis, with an average estimate for all patients from 70% to 75% (Ganesh et al., 2019); our patients with Crouzon (50%), SCS (25%), and AS (33%), presented strabismus.

Comparison of the clinical features of the patients analyzed with literature was problematic, since each author defines their own classification of these syndromes, some are based on the shape of the skull, while others on the affected sutures, some include the extremities, and others take the variant into account. Then, it is convenient to review the original description of each syndrome, to define the main clinical characteristics that were considered when these syndromes were detected for the first time.

Table 2 summarizes the main clinical and molecular characteristics of our patients, compared with the description of the literature (Ciurea & Toader, 2009; Wilkie et al., 2010; Johnson & Wilkie, 2011; Agochukwu, Solomon, & Muenke, 2012; Palafox, Ogando‐Rivas, Herrera‐Rodríguez, & Queipo, 2012; Twigg & Wilkie, 2015; Ko, 2016 and Kutkowska‐Kaźmierczak, Gos, & Obersztyn, 2018).

Mantilla‐Capacho, Arnaud, Díaz‐Rodríguez, & Barros‐Núñez, 2005, cited that the AS has the upper limbs more affected than the lower extremities and described the polydactyly of the hands and feet, and this is similar in our patients. Lajeunie, Bonaventure, El Ghouzzi, Catala, & Renier, 2000, declared that hydrocephalus in Crouzon is a serious complication that occurs in 30% of patients; however, we only observed this in 33% of patients with AS, but not in Crouzon. Agenesis of the corpus callosum and malformations of limbic structures are described in patients with AS. de León et al. in 1987 mentioned that 6 of 10 patients with AS had partial or total agenesis of the corpus callosum. These anomalies could be important in the contribution of the pathogenesis of intellectual disability, not only in AS but also in the other syndromes. In our patients with AS, 50% had agenesis of the corpus callosum and intellectual disability was present in only 17%. Crouzon is less prone to intellectual disability and only occurs in 3% (Kreiborg, 1981). In SCS, affected people often do not have intellectual disabilities. PS is associated with normal intelligence (type I), but types II and III have a serious intellectual disability. In AS, the intellectual ability is variable, and at least 50% of patients are affected with intellectual disability (Gorlin, Cohen, & Hennekam, 2001). In MS, it ranges from normal intellectual performance to mild disability. In our results, AS (17%), MS (50%), PS (25%), and Crouzon (5%) patients had intellectual disability.

Imaging studies in the AS, Crouzon, and PS have revealed abnormalities of the middle and inner ear, including the malformed and fused middle ear ossicles, atresia of the external auditory canal (Crouzon and PS), and atrophy of the tympanic membranes (Crouzon), to name a few (Desai et al., 2010; Orvidas, Fabry, Diacova, & McDonald, 1999; Zhou, Schwartz, & Gopen, 2009). In our patients, the syndromes that presented otitis media were AS (100%) and SCS (25%), and hearing loss AS (17%) and Crouzon (10%). This latter pathology is related to speech disorders that in AS showed 17% and in Crouzon 5%. The literature mentions that hearing loss is a specific feature in MS, however, in our study these patients did not present this pathology.

The skin manifestations in AS are acne on the face, chest, back, upper arms, and hyperhidrosis, the latter is described as present in all AS patients (Cohen & Kreiborg, 1995). However, our results show that only 83% of AS patients presented it.

The advance in molecular biology in the last 20 years has allowed us to know more about craniosynostosis, and thus have the genotype–phenotype correlation. Nevertheless, this correlation in craniosynostosis has been difficult. One of the problems is the clinical differentiation of each syndrome, since most patients share certain phenotypic characteristics, whose expression is variable, which makes the definitive clinical diagnosis very difficult. Some authors describe cases with different clinical phenotypes caused by the same pathogenic variant or equivalent of the *FGFR1, FGFR2, and FGFR3* genes. Other authors describe that similar phenotype may be associated with different variants in the same or distinct gene, or may be due to incomplete penetrance of pathogenic variants in genes related to the disease (Passos‐Bueno, Sertié, Jehee, Fanganiello, & Yeh, 2008).

The frequency of p.Ser252Trp and p.Pro253Arg pathogenic variants for the *FGFR2* in AS has been reported in several populations: in Brazilians 59.25% and 37% (Passos‐Bueno et al., 1998), in Canadians 76% and 18% (Chun, Teebi, Azimi, Steele, & Ray, 2003), in French 97% and 3% (Lajeunie et al., 1999), in Japanese 83% and 16.66% (Sakai et al., 2001), in Taiwanese 87% and 13.33% (Tsai et al., 1999), in Thais 57% and 42.85% (Shotelersuk et al., 2003), and in Turkish 58% and 33% (Nur et al., 2014). These pathogenic variants in patients with AS of diverse ethnic groups also show a higher frequency in the p.Ser252Trp pathogenic variant, than in p.Pro253Arg pathogenic variant. In our patients, the frequency was also higher in p.Ser252Trp pathogenic variant (66%) than in p.Pro253Arg pathogenic variant (33%).

Several heterozygous pathogenic variants of *FGFR2* have been described in Crouzon, AS, and PS; however, these latter syndromes differ from Crouzon by their extremities and dermatological features (Kan et al., 2002). The syndactyly of the hands and feet is more common and severe in patients with the p.Pro253Arg pathogenic variant, whereas cleft palate, higher frequency of profound midface retrusion, and severe malocclusion are more frequent in patients with p.Ser252Trp pathogenic variant. In addition, strabismus and visual impairment are more frequent in the case of p.Ser252Trp pathogenic variant presence. Concerning to the clinical outcome, the prognosis of AS patients after craniofacial surgery was better in patients with p.Pro253Arg pathogenic variant than with p.Ser252Trp pathogenic variant (von Gernet, Golla, Ehrenfels, Schuffenhauer, & Fairley, 2000). In our results, one patient with the p.Pro253Arg pathogenic variant had severe syndactyly in the hands and feet, while a patient with the p.Ser252Trp pathogenic variant showed cleft lip/palate.

Moloney et al. (1997) mentioned that it is difficult to clinically distinguish MS patients with and without the pathogenic variant p.Pro250Arg. Most of the time, they are misdiagnosed and confused with SCS. In our study, the patient who presented the p.Pro250Arg pathogenic variant was also misdiagnosed as PS and the molecular diagnosis allowed confirmation of MS. On the other hand, another patient who was initially considered as MS, not presenting the pathogenic variant p.Pro250Arg, was misdiagnosed, since the diagnosis could not be clearly defined, either clinically or molecularly.

González‐del Angel et al. (2016) studied 56 patients with apparently nonsyndromic uni‐ or bicoronal craniosynostosis, to identify the frequency and clinical characteristics of MS in a cohort of Mexican children. In only 8 of 56 probands (14%), the p.Pro250Arg pathogenic variant was found. They also evaluated first‐degree relatives for the p.Pro250Arg pathogenic variant and those resulted heterozygous underwent complete clinical examination. The results obtained were as follows: 7 of 20 relatives had the pathogenic variant, which looked apparently normal, however, when more detailed clinical and imaging tests were performed, alterations compatible with MS were found. In a family of MS, a heterozygous male brother p.Pro250Arg pathogenic variant presented hydrocephalus without craniosynostosis as the only clinical manifestation, whose characteristic had not been previously detected. Therefore, the authors suggest that some patients with MS may present only this manifestation as an expansion of the MS phenotype, rather than an unrelated finding. In conclusion, the authors mention that all patients with coronal craniosynostosis should be tested for the pathogenic variant p.Pro250Arg in the *FGFR3* gene, to confirm the diagnosis of MS.

**FIGURE 2:**
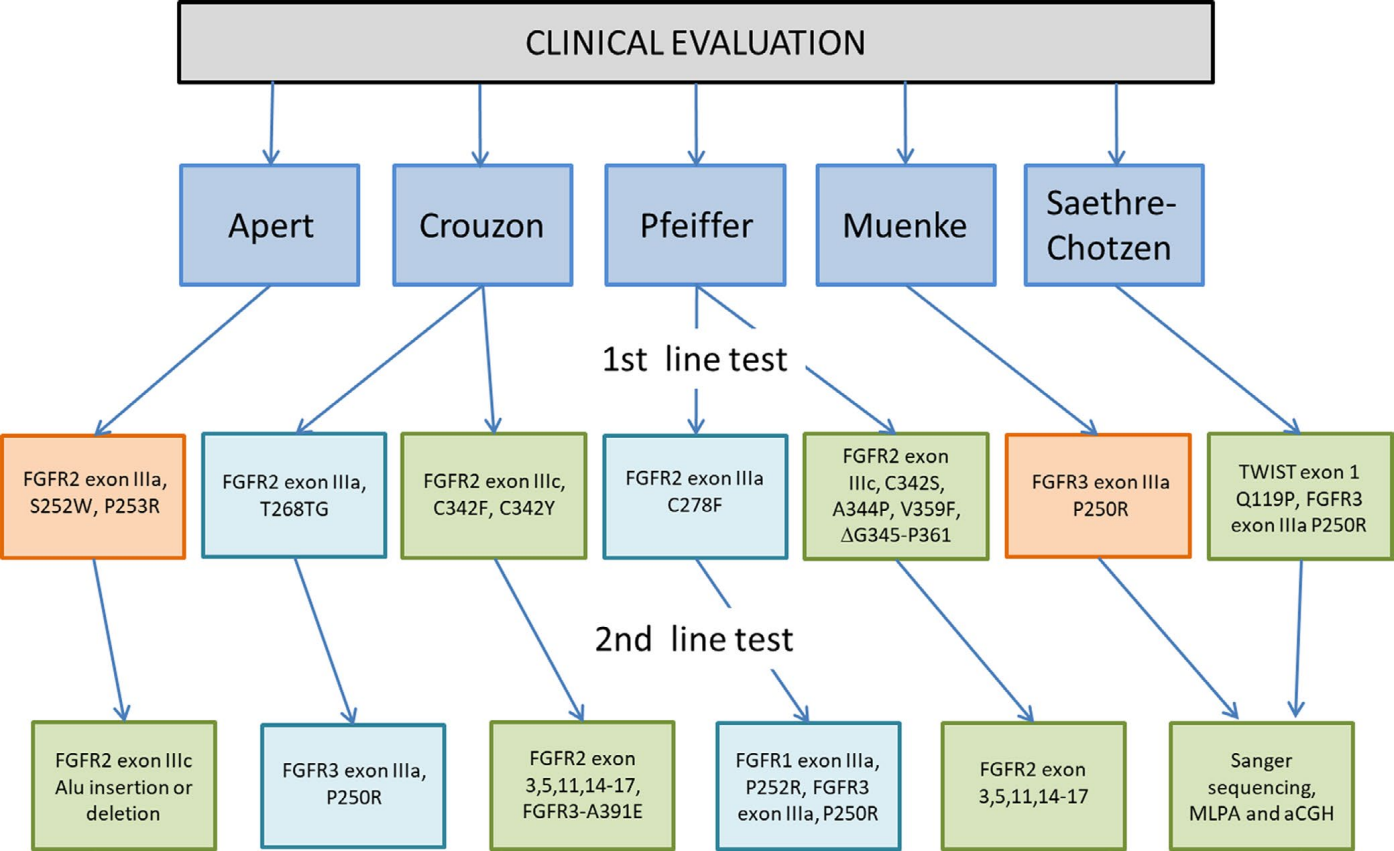
Molecular strategy for the analysis of patients with syndromic craniosynostosis, using the *FGFR1*, *FGFR2*, *FGFR3,* and *TWIST1* genes. In the orange boxes the analyzed pathogenic variants that were presented in the patients are shown, the light blue boxes are the molecular analyzes that were performed and gave negative, and the green boxes are the analyzes that should be done to confirm each syndrome, but that were not carried out in this study. MLPA, multiplex ligation‐dependent probe amplification; aCGH, array comparative genomic hybridization. Adapted from Johnson and Wilkie (2011) and Kutkowska‐Kaźmierczak et al. (2018)

Given the complexity that occurs in syndromic craniosynostosis, we decided to elaborate Figure 2 to show the strategy used in this study, considering the pathogenic variants most frequently analyzed as adjuvants in clinical diagnosis. In this sense, the opinion of other authors is that clinical evaluation is a fundamental part of the diagnosis and should be performed rigorously. Once the clinical diagnosis is established, the minimum molecular tests to confirm the diagnosis are described in the first and second line of the flow diagram. Other working groups recommend that if none of the most frequent pathogenic variants are found in patients, the following steps are as follows: the review of the clinical diagnosis, the search for other pathogenic variants in the genes analyzed, and in other genes involved in the pathogenesis of the disease, for example, *TCF12* (OMIM 600,480), *MSX2* (OMIM 123,101), *RAB23* (OMIM 606,144), and *EFNB1* (OMIM 300,035), using different methodologies such as multiplex ligation‐dependent probe amplification (MPLA), array comparative genomic hybridization (aCGH), and next‐generation sequencing (NGS).

In conclusion, although enormous advances have been made in recent years in the detection and analysis of the genes responsible for craniosynostosis, the great heterogeneity represented by this disorder continues to be a complex challenge, which requires long‐term studies to identify others disease‐related genes, as well as the inclusion of different populations.

## 5 LIMITATIONS OF THE STUDY

1. The small size of the sample and the number of pathogenic variants studied.
2. Two patients, who had many clinical features that pointed to a diagnosis of Crouzon, were misdiagnosed, since they presented alterations of hands and feet, but the specific pathogenic variant was not performed. In the same way, a patient, who was thought to have MS, was also ruled out as such, since he did not present the characteristic pathogenic variant.
3. Another limitation was that we did not have MRI or CT studies of all patients.
4. The molecular strategy used did not include all the pathogenic variants necessary for the analysis of patients with syndromic craniosynostosis (see Figure 2). These limitations could be solved in the future, performing tests that were not included in the study, in addition to carrying out multiplex ligation‐dependent probe amplification, array comparative genomic hybridization and sequencing, in those patients who are negative for known pathogenic variants.

However, despite these limitations, we consider that our study could be a contribution to the knowledge of these pathologies in Mexicans.

## 6 CONFLICT OF INTEREST STATEMENT

The Authors declare no conflict of interest.

## AUTHOR’S CONTRIBUTIONS

All authors prepared the bibliographic survey, manuscript preparation, and approved the final version of the manuscript.

## Data Availability

Data available upon reasonable request.
